# Association between problematic internet use and behavioral/emotional problems among Chinese adolescents: the mediating role of sleep disorders

**DOI:** 10.7717/peerj.10839

**Published:** 2021-02-22

**Authors:** Wanxin Wang, Xueying Du, Yangfeng Guo, Wenyan Li, Sheng Zhang, Lan Guo, Ciyong Lu

**Affiliations:** 1 Department of Medical Statistics and Epidemiology, School of Public Health, Sun Yat-Sen University, Guangzhou, China; 2 Health Promotion Centre for Primary and Secondary Schools of Guangzhou Municipality, Guangzhou, China

**Keywords:** Sleep disorders, Emotional problems, Behavioral problems, Adolescents, Problematic Internet use

## Abstract

**Background:**

Studies that focus on the relationships of problematic Internet use (PIU), sleep disorders, and behavioral/emotional problems were limited. This study aimed to explore (1) the relationship between PIU and behavioral/emotional problems among Chinese adolescents and (2) whether sleep disorders mediate the relationship between PIU and behavioral/emotional problems.

**Methods:**

A total of 1,976 adolescents were recruited by cluster sampling from ten secondary schools in Guangzhou between January and April 2019, and 1,956 of them provided valid information (response rate: 98.9% ). Among them, 50.8% were males and the mean age was 13.6±1.5 years, ranging from 11 to 18 years. Data on behavioral/emotional problems, sleep disorders, and PIU were collected using a self-reported questionnaire. Linear regression models and mediation analyses were performed.

**Results:**

Of the participants, 14.5% (284/1,956) reported moderate to severe PIU, and their average score for total difficulties was significantly higher than the score for average users (14.9±5.5 Vs 9.8±4.7). After adjusting for controlled variables, PIU was further proven to be positively related to elevated levels of behavioral/emotional problems (unstandardized *β* = 0.16, *p* < 0.05). In addition, sleep disorders partially mediated the forgoing associations.

**Conclusions:**

Adolescents with problematic Internet habits were at higher risk of developing behavioral and emotional problems than their normal-use peers, and sleep disorders partially mediated the effect. Close attention and effective guidance for adolescents with PIU and behavioral/emotional problems were recommended for parents and schools.

## Introduction

With its rapid development in recent decades, the Internet has become an indispensable part of people’s lives. However, while providing people with great convenience in many areas, the Internet can also lure people into problematic use and addiction. In recent years, the prevalence of problematic Internet use (PIU) has increased dramatically (Carbonell et al., 2018). A large amount of information and services provided on the Internet has dramatically supported people in their lives. In adolescence, a sensitive period in which self-restraint has not been fully developed, the Internet may be particularly tempting with its massive amount of information and novel content. PIU has become a serious health issue worldwide in recent years (WHO, 2015). Large-scale investigations in China have indicated that the prevalence of Internet addiction among adolescents was as high as 15% (Yang et al., 2018), with 2.2% reporting severe addiction (Guo et al., 2018). Certain Internet activities, such as online gaming and gambling, may negatively influence adolescents; for example, causing social problems and dysfunctional family relationships (Torres-Rodriguez et al., 2018) and directly or indirectly causing mental disorders (e.g., depression, anxiety, sleep disturbances, behavioral and emotional disorders), especially for individuals with PIU (Lam, 2014). Evidence has indicated that PIU is one of the major contributors to behavioral/emotional problems among adolescents (Tsitsika et al., 2014).

Behavioral and emotional problems have become a major mental health issue among adolescents in recent years, with 10–20% of adolescents experiencing mental disorders worldwide (WHO, 2019). Behavioral/emotional problems can emerge at a very young age, with an upward trend from age five and a significant increase during adolescence (Patalay & Fitzsimons, 2017). Studies have indicated that behavioral/emotional problems may lead to major depressive disorders (McCann et al., 2014), self-injury, substance abuse (Wilens et al., 2013), and suicidal behaviors (Guo et al., 2019). However, to establish effective interventions at an early stage, it is critical first to identify the risk factors and mechanisms underlying behavioral/emotional problems.

It is well established that sleep disorders are comorbid with and are significant predictors of psychiatric disorders such as internalizing and externalizing problems and suicidal behaviors (Guo et al., 2018; Lovato & Gradisar, 2014; Reynolds & Alfano, 2016). In addition, sleep disorders are among the major consequences of PIU (Yang et al., 2018). Epidemiological evidence from a systematic review demonstrated a positive relationship between PIU and sleep disorders, including insomnia, short sleep duration, and suboptimal sleeping quality (Lam, 2014). Screen light emitted from electronic devices can affect sleeping quality by altering people’s circadian rhythm and hormone levels, especially at night (Cain & Gradisar, 2010; Lemola et al., 2015). The potential pathways linking PIU to behavioral/emotional problems have not been fully discovered. There is evidence demonstrating the indirect effect of sleeping quality on the association between excessive Internet use and mental disorders among adolescents (An et al., 2014; Guo et al., 2018). However, limited studies focus on sleep disorders as mediators of PIU and behavioral/emotional problems among Chinese adolescents. As a result, the current study aimed to explore (1) the associations among PIU, sleep disorders, and behavioral/emotional problems and (2) the mediating role of sleep disorders in PIU and behavioral/emotional problems.

## Materials and Methods

### Study design and participants

Cross-sectional data were drawn from the baseline part of the 1-year Longitudinal Study of Adolescents’ Mental and Behavioral Well-being in Guangzhou, China between 2019 and 2020 (Registration No. ChiCTR1900022032). Ten public high schools from four major districts in Guangzhou were sampled using cluster sampling, with the school department as the sampling unit. All the 1st graders and their legal guardians were sent an informed consent letter explaining the intention of this study, and those who agreed to participate were included. Self-report questionnaires were distributed in classrooms with a trained researcher helping participants with their questionnaires and checking the rationality of the answers. In order to protect participants’ privacy and avoid information bias, the questionnaires and data analyses procedure were completely de-identified and anonymous. A total of 1,976 participants were included, and 1,956 (response rate: 98.99%) were eligible to participate and completed the questionnaire. A total of 50.8% of the participants were boys, and the age ranged from 11 to 18 years, with an average age of 13.6 (±1.5) years.

### Dependent variables

Behavioral and emotional problems were assessed using the self-report Strengths and Difficulties Questionnaire (SDQ). This questionnaire was first designed and validated by Goodman to evaluate young people’s mental health (Goodman, 1997). There are 25 items assessing participants’ hyperactivity, emotional symptoms, conduct problems, peer problems and prosocial behaviors. The total score for hyperactivity, emotional symptoms, conduct problems, and peer problems indicates the total difficulties score ranging from 0 to 40 (Goodman, 1997). A higher score indicates more behavioral/emotional disorders. In addition, a higher score of the prosocial subscale indicated more prosocial behaviors. Cronbach’s alpha for the SDQ were 0.76 and 0.74 for the total difficulties in the present study. The Chinese version was validated in a previous study (Liu et al., 2013).

### Independent variables

PIU was assessed by the Chinese version of the Young’s Internet Addiction Test (IAT), which consists of 20 questions related to whether and how the Internet use affects young people’s daily lives. The total IAT score ranges from 20 to 100, and a higher score indicates more severe symptoms of PIU. The validated cut-off point criteria proposed by Young were 20–49 (average users), 50–79 (moderately addicted) and 80-100 (severely addicted). In the present study, both the total IAT score and a cut-off point of 50 were adopted. The Cronbach’s alpha was 0.90 in the present study.

The presence of sleep disorders was assessed using the Chinese version of the Pittsburgh Sleep Quality Index (PSQI), which has been widely used to measure sleep quality and disorders. The Chinese version was validated by Tsai and colleagues (Tsai et al., 2005), and the PSQI consists of 18 items and 7 components: sleep quality, sleep duration, sleep efficiency, sleep latency, sleep disturbance, use of sleep medication, and daytime dysfunction. The global score ranges from 0 to 21, and a higher score indicates more severe sleep disorders. The Cronbach’s alpha was 0.77 in the present study.

### Demographic variables

Sex (1 = male, 2 = female), age, ethnicity (1 = Han, 2 = Other), family economic status, family relationships, and perceived academic pressure were adjusted as categorical variables with dummy variables set, except for age, in multivariable analyses. Family economic status was coded as 1 = above average, 2 = average, and 3 = below average, representing participants’ perception of their family’s financial status. Family relationships were measured by asking whether the relationships among family members were 1 = harmonious, 2 = average or 3 = conflicted. Perceived academic pressure was assessed by adolescents’ perception of academic pressure at school: 1 = mild, 2 = moderate, or 3 = heavy. The school unit was also adjusted as a continuous variable in the models.

### Statistical analyses

R language 3.6.0 and SPSS 25.0 were adopted for the statistical analyses. Baseline characteristics were described by the Mean ± SD for the continuous variables and the number and proportions for the categorical variables. Univariable and multivariable linear regression analyses were conducted for the relationship between the demographic covariates, PIU, sleep disorders, and behavioral/emotional problems. Demographic variables and covariates that were significant at the 0.25 level or widely reported by previous studies were further submitted to the multivariable analysis (Hosmer, Lemeshow & Sturdivant, 2013). Moreover, mediation analyses based on the methods proposed by Preacher and Hayes (Preacher & Hayes, 2004) were conducted using PROCESS 3.0 in SPSS, with the 5000 bootstrap samples for bias correction and 95% confidence interval. All the tests were two-tailed and the *p*-value< 0.05 was considered significant.

### Ethics

The study received approval from Sun Yat-sen University, School of Public Health Institutional Review Board (Ethics Approval Number: L2017060, date obtained: 2017/12/08). All the invited participants received a consent letter with the intention of the current study fully described. Consent letters were sent to legal guardians for participants under 18 years old. Participation was entirely voluntary.

**Table 1:** Baseline characteristics of the participating adolescents (*n* = 1, 956).

Variables	Total	Behavioral/emotional problems (M ±SD)	
	*n* (%)	Total difficulties	Prosocial behaviors
Age (years)	13.6 ± 1.5		
Sex			
Boys	993 (50.8)	10.2 ± 4.9	6.8 ± 2.3
Girls	963 (49.2)	10.8 ± 5.3^*^	7.5 ± 2.0^*^
Ethnicity			
Han	1,912 (97.8)	10.5 ± 5.1	7.2 ± 2.2
Other	44 (2.2)	11.1 ± 6.4	7.1 ± 2.1
Economic status			
Above average	1,012 (51.9)	9.8 ± 4.8	7.4 ± 2.2
Average	861 (44.2)	11.1 ± 5.2^*^	7.0 ± 2.2^*^
Below average	77 (3.9)	13.6 ± 5.7^*^	6.7 ± 2.2^*^
Missing	6		
Family relationship			
Harmonious	1,659 (85.2)	9.9 ± 4.8	7.3 ± 2.2
Average	230 (11.8)	13.4 ± 5.5^*^	6.6 ± 2.4^*^
Conflicted	59 (3.0)	16.4 ± 5.2^*^	7.0 ± 2.1
Missing	8		
Academic pressure			
Mild	515 (26.4)	9.2 ± 4.6	7.3 ± 2.2
Moderate	910 (46.6)	10.1 ± 4.8^*^	7.1 ± 2.2
Heavy	526 (27.0)	12.6 ± 5.5^*^	7.2 ± 2.2
Missing	5		
Behavioral/Emotional problems (M ± SD)			
Total difficulties	10.5 ± 5.1	–	–
Conduct problems	2.1 ± 1.5	–	–
Peer problems	3.0 ± 1.6	–	–
Hyperactivity	3.2 ± 2.1	–	–
Emotional problems	2.3 ± 2.2	–	–
Prosocial behaviors	7.2 ± 2.2	–	–
Problematic Internet Use			
M ± SD	36.5 ± 12.7	–	–
Average users	1,672 (85.5)	9.8 ± 4.7	7.2 ± 2.2
Problematic users	284 (14.5)	14.9 ± 5.5^*^	6.8 ± 2.3^*^
Sleep Disorders (M ± SD)	5.3 ± 2.8	–	–

**Notes.**

* unadjusted univariable linear regression analyses *p* < 0.05.

Total difficulties: Total score generated from emotional problems, conduct problems, hyperactivity, peer problems.

Prosocial behaviors: a sub-scale in strength and difficulties questionnaire assessing young people’s positive social behaviors.

## Results

### Baseline characteristics and univariable analyses

As shown in Table 1, a total of 1,956 participants were included in this study, with 993 (50.8%) boys and 963 (49.2%) girls. The age ranged from 11 to 18 years old, with a mean of 13.6 ± 1.5 years. The mean PIU score was 36.5 ± 12.7, with 14.5% of the participants reported as severe users. The average score for total difficulties in the SDQ was 10.5 ± 5.1, and the score for each subscale ranged from 2.1 to 3.2. We also observed significantly higher scores for total difficulties among individuals with PIU compared to average users (14.9±5.5 VS 9.8±4.7), while the result for the prosocial score was the opposite. The Pearson correlation analyses in Table 2 show that PIU and sleep disorders were significantly correlated with each other and that both were related to behavioral/emotional problems as well as to each subscale.

Table S1 demonstrated the baseline characteristics by sex. Age was similar between boys and girls. We can see that boys demonstrated a slightly higher score of behavioral and peer problems and lower prosocial behaviors scores. In comparison, girls showed a higher score of emotional problems, PIU as well as sleep disorders.

The unadjusted results in Table 3 and Table S2 showed that sex, family economic status, family relationships, academic pressure from school, PIU, and sleep disorders were linked to higher levels of behavioral/emotional problems. In addition, univariable analyses showed that PIU was positively related to sleep disorders (*β*=0.09 *p* < 0.05, Table 4).

**Table 2:** Pearson correlations between problematic Internet use, sleep disorders and behavioral/emotional problems.

	Problematic Internet Use	Sleep disorders
Problematic Internet Use	–	
Sleep disorders	0.40^*^	–
Total difficulties	0.46^*^	0.48^*^
Conduct problems	0.35^*^	0.25^*^
Peer problems	0.12^*^	0.16^*^
Hyperactivity	0.42^*^	0.38^*^
Emotional problems	0.36^*^	0.46^*^
Prosocial behaviors	−0.10^*^	−0.08^*^

**Notes.**

* *p* < 0.05.

### Adjusted associations among PIU, sleep disorders, and behavioral/emotional problems

As shown in Table 3, after adjustment for covariates, higher levels of PIU (*β* = 0.16, *p* < 0.05) indicated significantly more severe behavioral/emotional problems. Furthermore, the total difficulties score increased by 0.75 (*p* < 0.05) per one unit of the sleep disorders scale. The forgoing associations remained significant when sleep disorders and PIU were each entered into the models. Moreover, the association between PIU and sleep disorders was significant (*β* = 0.07, *p* < 0.05, Table 4) after adjusting for covariates.

**Table 3:** Associations between problematic Internet use, sleep disorders and behavioral/emotional problems.

Independent variables	Total difficulties	Conduct problems	Peer problems	Hyperactivity	Emotional problems	Prosocial behaviors
	Unstandardized *β* (SE)					
Problematic Internet Use						
Model 1^a^	0.19 (0.008)^*^	0.04 (0.002)^*^	0.01 (0.003)^*^	0.07 (0.003)^*^	0.06 (0.004)^*^	−0.02 (0.004)^*^
Model 2^b^	0.16 (0.008)^*^	0.04 (0.002)^*^	0.01 (0.003)^*^	0.06 (0.003)^*^	0.05 (0.004)^*^	−0.014 (0.004)^*^
Model 3^c^	0.12 (0.008)^*^	0.03 (0.003)^*^	0.006 (0.003)	0.05 (0.004)^*^	0.03 (0.004)^*^	−0.01 (0.004)^*^
Sleep disorders						
Model 4^a^	0.87 (0.037)^*^	0.13 (0.011)^*^	0.09 (0.013)^*^	0.28 (0.016)^*^	0.37 (0.016)^*^	−0.06 (0.018)^*^
Model 5^b^	0.75 (0.038)^*^	0.12 (0.012)^*^	0.08 (0.013)^*^	0.25 (0.016)^*^	0.31 (0.017)^*^	−0.06 (0.019)^*^
Model 6^d^	0.57 (0.038)^*^	0.07 (0.012)^*^	0.07 (0.014)^*^	0.18 (0.016)^*^	0.26 (0.017)^*^	−0.04 (0.020)^*^
Adjusted R^2e^	0.38	0.18	0.05	0.27	0.33	0.06

**Notes.**

a unadjusted *β* and standard error.

b adjusted for age, sex, ethnicity, school unit, family economic status, family relationship, academic pressure.

c adjusted for covariates in Model 1 and sleep disorders.

d adjusted for covariates in Model 5 and problematic Internet use.

e for model including problematic Internet use, sleep disorders, sex, age, ethnicity, school unit, family economic status, family relationship, academic pressure.

* *p* < 0.05.

**Table 4:** Associations between problematic Internet use and sleep disorders.

Problematic Internet use →Sleep disorders	Unstandardized *β* (SE)	Adjusted R^2^
Model 1^a^	0.09 (0.005)^*^	0.16
Model 2^b^	0.07 (0.005)^*^	0.22

**Notes.**

a unadjusted model.

b adjusted for age, sex, ethnicity, school unit, family economic status, family relationship, academic pressure.

* *p* < 0.05.

**Table 5:** Indirect effect of sleep disorders on problematic Internet use and behavioral/emotional problems.^a^

	Direct effect	Indirect effect
	*β* (95% CI)	
Total difficulties	0.12 (0.10, 0.13)^*^	0.04 (0.03, 0.05)^*^
Conduct problems	0.03 (0.03, 0.04)^*^	0.005 (0.003, 0.007)^*^
Peer problems	0.006 (−0.001, 0.01)	0.005 (0.003, 0.01)^*^
Hyperactivity	0.05 (0.040, 0.054)^*^	0.01 (0.010, 0.016)^*^
Emotional problems	0.03 (0.02, 0.04)^*^	0.02 (0.015, 0.023)^*^
Prosocial behaviors	−0.01 (−0.02, −0.004)^*^	−0.003 (−0.006, −0.0004)^*^

**Notes.**

* *p* < 0.05.

a adjusted for age, sex, ethnicity, school unit, family economic status, family relationship, academic pressure.

### Indirect effect of sleep disorders on PIU and behavioral/emotional problems

Mediation analyses indicated that sleep disorders partly mediated the association between PIU and behavioral/emotional problems. As shown in Table 5, the indirect effect of sleep disorders on the association between PIU and total difficulties was 0.04 (95%CI [0.03–0.05]). In addition, sleep disorders also partly mediated the association between PIU and conduct, emotional and hyperactive problems, and prosocial behaviors, respectively. However, when sleep disorders were entered into the linear regression model of PIU and peer problems, the direct effect of PIU was no longer statistically significant, implying a possibly complete indirect effect.

## Discussion

The average PIU score was 36.5 ± 12.7 in the current study, with 14.5% of the participants reporting problematic use. The mean global score for total behavioral and emotional difficulties was 10.5, with each difficulty subscale having a mean score between 2 and 3 and a score of 7 for the prosocial subscale. In addition, this study presented a positive association between PIU and behavioral/emotional problems, with sleep disorders mediating the aforementioned association.

The mean score for PIU was similar to that in former research, while the prevalence was slightly lower (Guo et al., 2018; Yang et al., 2018). The differences in the sample size and baseline characteristics of the participants may be the underlying contribution for such variance. The average levels for sleep disorders and behavioral and emotional difficulties in this study were similar to those reported in other studies in China (Guo et al., 2019; Xiao et al., 2019).

The univariable analyses suggested that sex, family economic status, family relationship, and perceived academic pressure were related to total behavioral and emotional difficulties and subscales. Girls were more vulnerable to having a higher level of total difficulties and emotional problems, while boys were more likely to report conduct and prosocial problems. Low economic status, conflicted family relationships, and heavy academic pressure were also associated with increased risks of behavioral and emotional difficulties. Previous studies also came to similar conclusions (Emam, Kazem & Al-Zubaidy, 2016; Wang, Liu & Wang, 2014).

The current study found that PIU was positively related to having more behavioral/emotional problems and negatively related to prosocial behaviors. For the subscales, we discovered that users with more severe PIU demonstrated higher levels of emotional, conduct, and peer problems as well as hyperactivity. Machimbarrena et al. (2019) also pointed out that PIU among adolescents was strongly related to lower levels of adolescents’ physical, psychological, and social and family well-being. Specifically, the authors found that PIU, especially severe use or Internet addiction, caused a serious loss of psychological health, including depressive and somatic symptoms, as well as behavioral/emotional problems among adolescents (Cerruti et al., 2017; Machimbarrena et al., 2019). It has been proposed that Internet addiction is usually related to people’s reward system, memory and cognitive ability and to the cerebral synaptic activity in the brain, which is subsequently linked to depression and behavioral disorders (Kuss, 2013). Among people with Internet gaming addiction, reduced dopamine transport has also been proven to be account for depression and some personality disorders (Bernardi & Pallanti, 2009; Morrison & Gore, 2010). Another possible explanation is that some inappropriate content on the Internet, such as aggressive or dishonest behaviors, plays a guiding role for adolescents to imitate, destructively influencing them. Fisoun et al. reported that abusive Internet use was significantly correlated with anti-social and aggressive behaviors among adolescents, giving rise to increasing behavioral problems (Fisoun et al., 2012). On the other hand, Yang et al. pointed out that compared to infrequent Internet users, frequent users were more likely to be emotionally unstable, affective, and lonely, which can lead to emotional issues among adolescents (Yang et al., 2005). Additionally, more harmful Internet activities were associated with worse interpersonal activities and fewer prosocial attributes (Ma, Li & Pow, 2011; Milani, Osualdella & Blasio, 2009), a finding that is consistent with the negative relationship between PIU and prosocial behaviors in the current study. Social withdrawal and isolation symptoms generated from excessive dependence on the Internet were found to be another pathway to psychiatric disorders and behavioral problems (Kato, Shinfuku & Tateno, 2020). Taken together, our results regarding the strong association of PIU with behavioral/emotional problems among adolescents are supported by previous studies. In particularly, our data demonstrated that compared to the other three problems, higher score of PIU caused more increase in hyperactivity and emotional problems, indicating that these problems were more likely be affected by inappropriate Internet use.

We also found that PIU was positively associated with sleep disorders, which were further related to elevated levels of behavioral/emotional problems. There is existing evidence supporting a strong relationship of PIU with sleep disorders (Yang et al., 2018), as well as evidence that suboptimal sleeping quality increases the likelihood of behavioral/emotional problems (Kosticova, Husarova & Dankulincova, 2020). In addition, we also found that sleep disorders caused more emotional, hyperactive problems, compared to other sub-scales. In conclusion, our data demonstrated that adolescents with a mean age of 13.5 were prone to developing hyperactive and emotional problems if they have inappropriate Internet use habits or sleep problems.

Furthermore, we identified a partial indirect effect of sleep disorders on the relationships between PIU and behavioral/emotional problems and prosocial behaviors in this study. Previous studies have illustrated that sleep situations may be a potential mechanism explaining the relationship between PIU and psychiatric disorders among both adolescents and adults (An et al., 2014; Kim et al., 2017). Internet use, especially excessive use or addiction, becomes increasingly challenging for people to abstain from. The withdrawal symptoms cause serious disturbances in people’s daily lives, including regular sleeping habits (Herlache, Lang & Krizan, 2018). Inadequate sleeping and circadian activities were proven to be associated with people’s psychiatric well-being via their hormone levels. Dysregulated cortisol and pubertal hormone levels, such as testosterone or estradiol, were detected among children and adolescents with sleeping and behavioral/emotional disorders (Dolsen, Deardorff & Harvey, 2019; Hatzinger et al., 2012). Our study also showed that the indirect effect of PIU on peer problems was statistically significant, while the direct effect was not, with the 95% CI of the mediated proportion covering 100%, suggesting a possibly complete mediation effect of sleep disorders. In the future study, we will further confirm this association in a longitudinal study with larger sample size.

Considering that Internet use is becoming an inevitable part of modern society, where adolescents pursue both their academic as well as entertainment goals, we suggested that parents and educational facilities should pay more attention to adolescents’ Internet use, including time and content, to prevent the development of problematic use and even addiction. Additionally, schools and educational facilities are advised to assign homework and after-school curricula relying more on real books and classrooms instead of online methods, thus reducing adolescents’ time spent on the Internet and electronic devices. In addition, the negative effect of Internet use on sleeping cannot be ignored. It is essential for students to build a healthy schedule of sleep and work, especially for Chinses students facing heavy academic pressure. Last but not least, adolescents’ psychological and behavioral well-being, as one of the major negative consequences of PIU and sleep disorders, should be a top priority during their development. Our study especially indicated that adolescents are more likely to develop hyperactive and emotional problems if exposed to too much Internet use or sleep problems during this period. Parents and teachers are strongly suggested to closely keep track of adolescents’ emotional and behavioral changes and provide counseling in a timely manner.

There were some limitations of this study that should be noted. First, the current study was the baseline part of a longitudinal study. As a result, it is a cross-sectional design, which made it impossible to draw causal conclusions. Results from this study are a preliminary exploration of the associations among PIU, sleep disorders and behavioral/emotional problems. We will further test the relationships with better time sequence through longitudinal study design in our future study. Second, the sample was gathered only in Guangzhou, a well-developed region of China. Moreover, the proportion of junior high school students was more extensive than that of senior high school students (72.6% vs. 27.4%), thus introducing selection bias. As a result, the conclusions from this study should be generalized with caution. Third, although self-reported questionnaire has been proven to be a valid research instrument, it may still generate information bias. On the other hand, this study has several strengths, one of which was providing a high level of quality control during the field survey. Trained interviewers were present to guide students with their questionnaires, which aimed to reduce some of the reporting bias. Another strength was that the school-based sample in this study ensured a high response rate and good cooperation from participants, thereby ensuring data quality.

## Conclusions

The current study suggested that PIU was positively related to adolescents’ behavioral and emotional problems, especially hyperactivity, emotional and conductive problems. Furthermore, sleep disorders partially mediated the effect of PIU on behavioral as well as emotional problems. This finding provided a possible mechanism of the effect of sleep disorders on the forgoing associations. Despite the convenience brought by the Internet, inappropriate use significantly disturbs adolescents’ sleep quality and further worsens their behavioral and emotional well-being. As a result, parents are highly encouraged to increase their attention to adolescents’ Internet use and alterations in mood and behavior and provide guidance when necessary. Moreover, a healthy sleeping schedule should be guaranteed for adolescents since sleep can affect their daily performance and their psychological and behavioral well-being.

##  Supplemental Information

10.7717/peerj.10839/supp-1 Supplemental Information 1 Adolescents’ raw data

10.7717/peerj.10839/supp-2 Supplemental Information 2 Data code and instruction

10.7717/peerj.10839/supp-3 Supplemental Information 3 Baseline characteristics of the participating adolescents by sex

10.7717/peerj.10839/supp-4 Supplemental Information 4 Unadjusted association between covariates and behavioral/emotional problems

## Additional Information and Declarations

### Data Availability

The following information was supplied regarding data availability: The raw measurements are available in the Supplementary Files.
