# Supplementary material for: Association between problematic internet use and behavioral/emotional problems among Chinese adolescents: the mediating role of sleep disorders
Source: PeerJ. 2021 Feb 22;9:e10839. doi: 10.7717/peerj.10839 (PMC7906038; doi:10.7717/peerj.10839)
Supplement: Supplemental Information 2 [file peerj-09-10839-s002.doc]

**Data instruction**

SchoolNo: code of each school the participants come from

a03Sex: Sex of the participants

a04Ethicnity: Ethnicity of the participants

a06: Economic status of the participants

a10: Family relationship of the participants

a14: Perceived academic stress of the participants

Sleep quality: c13-c20

Strengths and difficulties questionnaire: c2101-c2125

Problematic internet use: c2501-c2520
