# Supplementary material for: Association between problematic internet use and behavioral/emotional problems among Chinese adolescents: the mediating role of sleep disorders
Source: PeerJ. 2021 Feb 22;9:e10839. doi: 10.7717/peerj.10839 (PMC7906038; doi:10.7717/peerj.10839)
Supplement: Supplemental Information 3 [file peerj-09-10839-s003.docx]

| **Supplemental table 1** Baseline characteristics of the participating adolescents by sex (n=1,956) | | |
| --- | --- | --- |
| Variables | Boys (n=993) | Girls (n=963) |
|  | M ± SD | |
| Age (years) | 13.6±1.5 | 13.5±1.4 |
| Behavioral/Emotional problems |  |  |
| Total difficulties | 10.2±4.9 | 10.8±5.3 |
| Conduct problems | 2.1±1.5 | 2.0±1.4 |
| Peer problems | 3.0±1.6 | 2.9±1.6 |
| Hyperactivity | 3.3±2.1 | 3.2±2.1 |
| Emotional problems | 1.8±2.0 | 2.7±2.4 |
| Prosocial behaviors | 6.9±2.3 | 7.5±2.1 |
| Problematic Internet Use | 36.1±12.5 | 36.9±12.9 |
| Sleep Disorders | 5.2±2.9 | 5.5±2.7 |
