# Supplementary material for: Association between problematic internet use and behavioral/emotional problems among Chinese adolescents: the mediating role of sleep disorders
Source: PeerJ. 2021 Feb 22;9:e10839. doi: 10.7717/peerj.10839 (PMC7906038; doi:10.7717/peerj.10839)
Supplement: Supplemental Information 4 [file peerj-09-10839-s004.docx]

| **Supplementary table 2** Unadjusted association between covariates and behavioral/emotional problems | | | | | | |
| --- | --- | --- | --- | --- | --- | --- |
|  | Total difficulties | Conduct problems | Peer problems | Hyperactivity | Emotional problems | Prosocial behaviors |
|  | Unstandardized *β* (SE) | | | | | |
| **Sex** (Ref.=Boys) | 0.62 (0.23) * | -0.14 (0.07) * | -0.13 (0.07) | -0.06 (-0.09) | 0.94 (0.10) * | 0.67 (0.10) * |
| **Age** | 0.01 (-0.08) | -0.07 (0.02) * | 0.03 (0.02) + | -0.01 (0.03) | 0.03 (0.03) | 0.01 (0.03) |
| **Ethnicity** (Ref.=Han) | 0.64 (0.78) | 0.21 (0.22) | -0.40 (0.24) + | 0.72 (0.32) * | 0.06 (0.34) | -0.11 (0.34) |
| **Family economic status** (Ref.= Above average) | | | | | | |
| Average | 1.34 (0.23) * | 0.06 (0.07) | 0.26 (0.07) * | 0.46 (0.10) * | 0.54 (0.10) * | -0.39 (0.10) * |
| Below average | 3.79 (0.59) * | 0.69 (0.17) * | 0.75 (0.18) * | 1.35 (0.24) * | 1.00 (0.26) * | -0.66 (0.26) * |
| **Family relationship** (Ref.=Good) | | | | | | |
| Average | 3.51 (0.35) * | 0.65 (0.10) * | 0.47 (0.11) * | 1.30 (0.14) * | 1.07 (0.15) * | -0.70 (0.15) * |
| Conflict | 6.53 (0.65) * | 1.33 (0.19) * | 0.71 (0.21) * | 1.86 (0.27) * | 2.64 (0.29) * | -0.26 (0.29) |
| **Academic pressure** (Ref.=Mild) | | | | | | |
| Moderate | 0.86 (0.27) * | -0.08 (0.08) | 0.07 (0.09) | 0.46 (0.11) * | 0.41 (0.12) * | -0.15 (0.12) + |
| Heavy | 3.37 (0.31) * | 0.35 (0.09) * | 0.28 (0.10) * | 1.17 (0.13) * | 1.56 (0.13) * | -0.05 (0.14) |

* *p*<0.05

+ *p*<0.25
